# Assessing causal relationships in genomics: From Bradford-Hill criteria to complex gene-environment interactions and directed acyclic graphs

**DOI:** 10.1186/1742-7622-8-5

**Published:** 2011-06-09

**Authors:** Sara Geneletti, Valentina Gallo, Miquel Porta, Muin J Khoury, Paolo Vineis

**Affiliations:** 1 Department of Statistics, London School of Economics, Houghton Street, London, UK; 2 Department of Epidemiology and Public Health, Imperial College, London, UK; 3 Department of Social and Environmental Research, London School of Hygiene and Tropical Medicine, UK; 4 Institut Municipal d'Investigació Mèdica, and School of Medicine, Universitat Autònoma de Barcelona, Catalonia, Spain; 5 National Office of Public Health Genomics, Centers for Disease Control and Prevention, Atlanta, USA; 6 Department of Epidemiology and Public Health, Imperial College, London, UK; 7 HuGeF, Via Nizza 52, 10126 Torino, Italy

## Abstract

Observational studies of human health and disease (basic, clinical and epidemiological) are vulnerable to methodological problems -such as selection bias and confounding- that make causal inferences problematic. Gene-disease associations are no exception, as they are commonly investigated using observational designs. A rich body of knowledge exists in medicine and epidemiology on the assessment of causal relationships involving personal and environmental causes of disease; it includes seminal causal criteria developed by Austin Bradford Hill and more recently applied directed acyclic graphs (DAGs). However, such knowledge has seldom been applied to assess causal relationships in clinical genetics and genomics, even in studies aimed at making inferences relevant for human health. Conversely, incorporating genetic causal knowledge into clinical and epidemiological causal reasoning is still a largely unexplored area.

As the contribution of genetics to the understanding of disease aetiology becomes more important, causal assessment of genetic and genomic evidence becomes fundamental. The method we develop in this paper provides a simple and rigorous first step towards this goal. The present paper is an example of integrative research, i.e., research that integrates knowledge, data, methods, techniques, and reasoning from multiple disciplines, approaches and levels of analysis to generate knowledge that no discipline alone may achieve.

## Introduction

Observational studies of human health and disease (basic, clinical and epidemiological) are vulnerable to methodological problems -such as selection bias and confounding- that make causal inferences problematic. Gene-disease associations are no exception, as they are commonly investigated using observational designs. However, as compared to studies of environmental exposures, in genetic studies it is less likely that selection of subjects (e.g., cases and controls in a case-control study) is affected by genetic variants. Confounding is also less likely, with the exception of linkage disequilibrium (i.e., the attribution of a genetic effect to a specific gene rather than to an adjacent one) and population stratification (when cases and controls are drawn from different ethnic populations). There is in fact some empirical evidence suggesting that gene-disease associations are less prone to confounding (e.g., by socio-economic status) than associations between genes and environmental and lifestyle variables [1]. There are some well known methodological challenges in interpreting the causal significance of gene-disease associations; they include epistasis, linkage disequilibrium, and gene-environment interactions (GEI) [2].

A rich body of knowledge exists in medicine and epidemiology on assessment of causal relationships involving personal and environmental causes of disease; it includes seminal causal criteria developed by Austin Bradford Hill and more recently applied directed acyclic graphs (DAGs). Perhaps unsurprisingly, such knowledge has seldom been applied to assess causal relationships in clinical genetics and genomics, even when studies aimed at making inferences relevant for human health. Conversely, incorporating genetic causal knowledge into clinical and epidemiological causal reasoning is still a largely unexplored task.

In this paper, we first state our main aim; secondly, we propose applications of Hill's criteria to genetic problems and genetic epidemiology; thirdly, we use graphical methods to formulate and assess causal hypotheses involving genes; finally, we use a case study of Parkinson's disease to apply the combined Hill / DAGs approach to untangling the underlying GEIs.

## Aim of the paper

The main aim of this paper is to propose a conceptual framework to assess causal relationships in clinical genomics and, particularly, for evaluating the etiopathogenic significance of gene-disease associations and gene-environment interactions; i.e., a framework to assess the validity and significance of such environment-host-gene relationships in the etiology of human diseases. The framework includes a two-step approach that combines the causal criteria of Austin Bradford Hill with graphical models such as directed acyclic graphs (DAGs). The approach we propose thus helps, first, to untangle the web of interactions amongst several exposures and characteristics (environmental, clinical and genetic) and a disease. Once these relationships have been specified, they are analyzed using criteria to assess causality that have long been used in clinical and epidemiological research. More generally, the present paper is an example of *integrative research*, i.e., research that integrates knowledge, data, methods, techniques, and reasoning from multiple disciplines, approaches and levels of analysis to generate knowledge that no discipline alone may achieve [3].

## Applying causal guidelines to genetic studies

For several decades, guidelines to assess causality have been a powerful tool in clinical and epidemiological research, as well as in the professional practice of medicine and epidemiology outside academia [4-7]. Causal guidelines usually include a series of criteria that help assess which observed associations are potentially causal. They were introduced initially by Bradford-Hill in the debate about the role of smoking in the aetiology of lung cancer; given the issue, they were meant for observational studies only, but many of the criteria can be applied to clinical trials and other experimental studies as well [8]. Although Hill did not have genetic epidemiology in mind at the time, today his criteria remain relevant to causal assessment in this field and, as we will show, to many areas of human genetics as well.

Hill's approach is based on nine criteria: 1) Strength of association; 2) Consistency; 3) Specificity of association; 4) Temporality; 5) Biological gradient (dose-response relationship); 6) Biological plausibility; 7) Coherence; 8) Experimental evidence (e.g. reproducibility in animal models); and 9) Analogy. Statistical significance was not listed but discussed separately by Hill [8].

One major criticism leveled at Hill's approach is that it considers one causal factor at a time and is not intended to tackle complex relationships and interactions, such as those encountered in modern molecular medicine and genomics, which deal with chains of mediators and not only directly acting exposures. However, even complex situations can often be decomposed into simpler constituents, and in such case Hill's criteria can be applied fruitfully. This is a main motivation behind the present work.

In 2006, a Human Genome Epidemiology Network (HuGENet) workshop in Venice was devoted to the development of standardized criteria for the assessment of the credibility of cumulative evidence on gene-disease associations. This led to synopses on various topics in genetic epidemiology; e.g., on DNA repair [9], and on Parkinson's disease [10]. Briefly, according to the *Venice guidelines *[2] each gene-disease association is graded on the basis of the amount of evidence, replication, and protection from bias. These guidelines contributed to modifying the approach to genetic inferences using Hill's criteria that we adopt here.

Main theoretical issues underlying the application of Hill's criteria in genetics and genomics are shown in Appendix 1 [11-29]; below we will show how these criteria can be applied to an example of gene-environment interaction. Interactions here are defined as "the interdependent operation of two or more causes to produce, prevent, or control an effect" [2].

In summary, Hill's causal criteria and related logical tools that have long been applied fruitfully to clinical and epidemiological research may also be applied productively to research in genetics. However, genetic research has fundamental differences from clinical and epidemiological research. For example, in genetics confounding can be the consequence of events that may not be directly addressed at the other levels, including haplotype blocks, allelic heterogeneity, overdominance, and epistasis [15]. Selection bias is more easily measurable in genomic studies, because we have the null hypothesis represented by Hardy-Weinberg equilibrium (HWE); i.e., we expect independent assortment of alleles in the population, whereas a similar reasoning cannot be applied to daily life exposures. Hardy-Weinberg equilibrium is based on assumptions of population genetics related to the lack of selection, inbreeding, migration; departure from HWE can thus point towards the possibility of gross bias (such as genotyping errors or selection bias).

Explicit guidelines for causal assessment are more popular in clinical and epidemiological research than in genetics [3,30]. The reasons for that have seldom been addressed. They are probably related to the different nature of the objects, factors, mechanisms and processes that we study at each level. However, genetic guidelines on causality do exist and, in fact, have interesting similarities with Hill's criteria: (a) linkage to a particular region of the human genome (LOD>3); (b) one or more independent mutations that are concordant with disease status in affected families (specificity, strength of association); (c) defects that lead to macrochanges in the protein (specificity, coherence); (d) putative mutations that are not present in a sample from a control population (specificity); or (e) presence of some other line of biological evidence (including expression, knockout data, etc.) [15]. Criteria (a), (b) and (c) refer to background knowledge. But it is in particular criterion (e) that supports the causal association by conferring coherence with previous knowledge [3,15].

## Directed acyclic graphs as tools to clarify associations and complex causal relationships

Directed acyclic graphs (DAGs) have a long tradition in science. They are a rigorous way of visualising complex systems, clarifying ideas, complementing the formulation of hypotheses, and guiding quantitative analyses. There has been much debate on the exact nature and roles of DAGs in the biomedical literature. The most widespread approach in the health sciences is the *causal DAG *approach promoted by Greenland, Robins, Hernán and colleagues [31-33], and the equivalent mathematical framework of counterfactuals [34]. In causal DAG approaches, the directed edges in a DAG represent causal relationships. Whilst the causal DAG framework is appealing and intuitive, we wish to draw attention to an alternative approach to causal inference, the Decision Theoretic Framework (DTF), which is based on a formal treatment of conditional independences (a non-graphical version of the 'd-separation criteria') [35]. Appendix 2 provides additional details on statistics and assumptions underlying the DTF [36-38]. This approach has recently become increasingly popular in epidemiology, in particular to assess the role of genes as instrumental variables for causal inference [39]. DTF retains the advantages of the causal DAG approach but overcomes some of its limitations. In particular, as DTF uses DAGs to describe the relationships between variables, it retains the capacity of DAGs to clearly and formally visualise complex systems. In contrast to the causal DAG approach where all directed edges are assumed to represent causal relationships, DTF takes a more conservative view where the edges represent statistical associations (and the lack of edges represents independence). Causality in DTF is viewed as external knowledge that can be added to the DAGs and allows some of the edges to be interpreted as causal. There are three reasons for this conservative viewpoint. The first is that it entails fewer assumptions about the existence and direction of causal relationships between variables. The second is that it is not necessary to include all possible causes or covariates in a DAG, only the variables of interest, making DTF more flexible than the causal DAG approach. The third is that when we perform a statistical analysis of observational data, we obtain measures of association (not causation) between variables. We explain this concept in more detail below.

A main problem when making causal inferences in clinical and epidemiological research is that most data are observational. This is also true for a substantial part of basic biomedical research. It is certainly an issue in human genetics, where there is usually no randomization (except in circumstances where Mendelian randomization can be applied [1,3,39,40]), and knowledge of the genetic pathways is tenuous or incomplete. In such circumstances we must be careful to distinguish *causal *relationships from *associations *resulting from unobserved biases or chance.

DAGs can still be used to make causal inference, but the causal element is an external assumption that needs to be explicitly incorporated into the DAG rather than implicit in the direction of an edge. We use a DAG to visualise complex associations, but when we only have observational data at our disposal, we must find other ways to assess *a) *whether a particular association is causal and not due to confounding or other bias, and *b) *what the direction of this association is.

**Figure 1:**
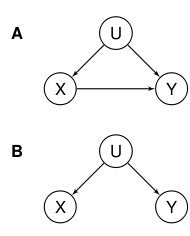
**DAG demonstrating the ideas of confounding**. A: U is an unobserved confounder for the association between X and Y and X is a cause of Y. B: U is an unobserved confounder for the association between X and Y but X is not a cause of Y. From purely observational data these two situations cannot be separated.

The problem of inferring causality from observational data in the presence of unobserved confounding is simply described in the DAGs in Figure 1.

**Figure 2:**
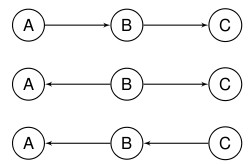
**Three DAGs exhibiting the same conditional independence but with different causal interpretations**.

**Figure 3:**
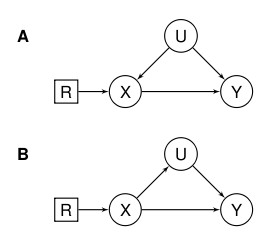
**DAG with a randomisation node R. R indicates whether X is randomised or allowed to arise naturally**. A: U is a confounder. B: U is a mediator. Randomisation allows us to distinguish between these situations.

In the DAG on the left hand side X is the putative cause -e.g., a particular environmental exposure such as urban pollution-, Y is the disease outcome under investigation, and U a set of confounders, many of which will typically be unobserved. Epidemiologists are interested in the existence, direction and strength of the X-Y association and whether this can be considered causal. (They are not necessarily interested in whether the other relationships in the DAG are causal). However, they are often unable to capture all this information from observational studies due to the presence of unobserved confounders U. Even when there is no direct association -i.e., there is no edge between X and Y as in the DAG on the right hand side of the Figure 1-, the presence of U (this time as a common parent) will result in a statistical association between the two. Again, the question is, how do we distinguish a causal association from a statistical association when only observational data are available?

One way to answer this question is by incorporating prior knowledge in Hill's scheme (or similar criteria) with DAGs to determine which edges can be considered causal. This is the approach we propose in this paper and that we describe in detail below. Another way of introducing causality is by adding so called *intervention *or *randomisation *variables to a DAG and to the corresponding probability statements. A more detailed description of such variables is given in Appendix 2. As a thorough explanation is beyond the scope of this paper we refer the interested reader to Dawid [41], Didelez [42], Geneletti [43], and Lauritzen [44].

For the remainder of this paper, the DAGs we use can be viewed as heuristic tools to understand gene-environment relationships.

## Parkinson's disease: pesticides, and gene-environment interactions

In order to illustrate our methods, we present a case study based on Parkinson's disease. First we present a short description of the disease and a summary literature review of its genetic component; we focus in particular on a recently identified genetic form. Second, we use graphical methods to propose and assess hypotheses on how the risk factors might interact. Third, we apply Hill's criteria to each of the hypothesised associations to assess causality in light of the available evidence.

Parkinson's disease is the most common neurodegenerative disorder after Alzheimer's disease, affecting 16-19 new individuals per 100,000 persons each year in developed countries [45]. Characterized by bradykinesia, resting tremor, rigidity and postural instability, it is also one of the most common late-life movement disorders. The pathological characteristic of the disease is a selective loss of pigmented neurons, most prominently in the *substantia nigra *(one of the brain basal ganglia) accompanied by a characteristic α-synuclein-positive inclusion bodies in neurons (Lewy bodies) [45]. While the causes of Parkinson's disease remain unknown, significant progress is being made in elucidating genetic and environmental risk factors and the neurodegenerative process underlying the disease. Appendix 3 summaries the key evidences to date on environmental and genetic risk factors for Parkinson's disease [46-49].

**Figure 4:**
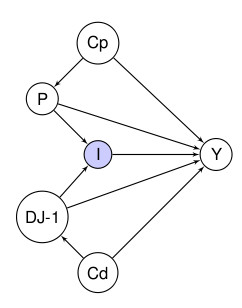
**DAG showing all possible one way relationships for gene-environment interactions based on the observed variables**.

A deletion of the *DJ-1 *gene in a Dutch family and a mutation conferring a functionally inactive form in an Italian family associated with early onset PD were first observed in 2001 [50], and confirmed in 2003 [51] (as is convention, we use italics to indicate the gene and non-italics to indicate the protein; thus, *DJ-1 *means the gene, and DJ-1 means the protein). DJ-1 is involved in many cell processes including oncogenic transformation, gene expression and chaperon activity, and it mediates oxidative stress responses [52]. A recent meta-analysis of the association between pesticides and Parkinson's disease [53] concludes that the epidemiologic evidence suggests a fairly consistent association between exposure to pesticides and risk of developing Parkinson's disease. In particular, among the herbicides, paraquat has been found to be most strongly associated with the risk of the disease (with odds ratios ranging from 1.25 to 3.22). Toxicological evidence suggests that both paraquat and rotenone exert a neurotoxic action that might play a role in the etiopathogenic process of Parkinson's disease. Moreover, clinical symptoms of Parkinson's disease have been reproduced in rats by chronic administration of paraquat [54]. Evidence from animal experiments shows that knockout models of Drosophila Melanogaster (fruit fly) lacking DJ-1 function, display a marked and selective sensitivity to the environmental oxidative insults exerted by both paraquat and rotenone [54]; this suggests that there is an interaction between these toxicants and the *DJ-1 *genotype [3]. On the basis of these data, it is sensible to hypothesise an interaction between *DJ-1*, exposure to some pesticides, and risk of Parkinson's disease in humans as well. Using Hill's criteria we can say that the hypothesis has biological plausibility; also, testing the hypothesis entails testing Hill's criterion of analogy (i.e., testing that there are analogous causal mechanisms in certain animal models and in humans). To test the hypothesis, further investigation is needed in order to estimate the effect of the interaction between *DJ-1 *and exposure to specific pesticides in humans on the risk of developing Parkinson's disease. We can construct a logic framework displaying (a) the association of paraquat (P) with Parkinson's disease (Y); (b) the association of *DJ-1 *with Parkinson's diseases; and (c) the interaction of *DJ-1 *with exposure to paraquat. We can also assume the existence of confounding between the exposure to paraquat and the disease outcome (Cp), and between *DJ-1 *and disease outcome (Cd) (Figure 4). First we are going to propose a graphical method to untangle the relationship between these two risk factors and Parkinson's disease; in a second step we will evaluate the associations from a more strictly causal point of view.

## Case study: the *DJ-1 *gene, exposure to paraquat and risk of Parkinson's disease

The process we describe in this section has two components. The first uses DAGs as a visual tool to explore a range of possible interaction scenarios. The second uses DAGs as a formal tool to describe the formal dependence among the variables in the problem. These two components go hand in hand, as intuition about the problem will generally guide the first whilst the second will reflect information in the observed data as well as considerations about what is biologically plausible. In a second instance, which is beyond the scope of this paper, the interaction quantitative effects can be estimated. How the latter step is done will depend both on the nature of the data available and crucially on the model for interaction. We assume an additive interaction model for simplicity; however, the DAGs work equally well with a multiplicative model as they describe associations rather than their exact mathematical nature.

We consider first the case study of gene-environment interactions (GEI) involving risk of Parkinson's disease, the *DJ-1 *gene and exposure to paraquat described above. To do this we use simplified versions of models proposed by Khoury et al. [55] and Ottman [56]. Subsequently, we consider fruit fly experiments where the associations between Parkinson's, *DJ-1 *and paraquat have been ascertained, and we present this as the ideal situation to make causal inference. The approach we are proposing can be also used to tackle a range of other complex problems.

In order to look at possible GEI scenarios we need to introduce some simple notation:

gene: *DJ-1 *= d* variant (deletion as in the Dutch families or inactivity as in the Italian families); *DJ-1 *= d wild type

pesticides: P = p* exposed; P = p unexposed

disease: Y = 1 with Parkinson's disease; Y = 0 without Parkinson's disease

The crux of this approach is the introduction of an interaction variable I. It is determined by the values of the genetic and environmental exposure variables. In simple terms, it acts like a switch and is turned "on" when the parents (a parent P of another variable X has an edge pointing into X, and X is a child of P) take on some values, and "off" when the parents have other values. In the current context this is typically the presence of the genetic exposure (i.e., the genetic variant) and/or the environmental exposure that leads to an increase in disease risk which turns the interaction "on". Thus, in addition to the above variables, we also define:

interaction: I = 1 ("on") if there is an interaction and I = 0 ("off") if there isn't. The exact nature of the interaction depends on the contexts sketched below.

For the sake of simplicity, we assume that I is a *deterministic *variable. What we mean by this is that unlike the other variables in the problem, I is not random. Once the value of its parents is known, then so is the value of I. This might be considered unduly restrictive if there are other potential parents in the interaction which are suspected but unobserved. It is possible in these cases to view I as a random variable, where its variability is associated with that of the unobserved interactant. However, in the paper we focus on the simplest case and thus we make the following assumption:

1. *DJ-1 *and P are the only parents of the interaction variable I. Another assumption that is generally plausible, provided that the exposure does not modify the genetic structure (e.g., the exposure does not cause somatic mutations) is that:

2. There is no *a priori *association between the gene and the external exposure; this is represented by the absence of a directed edge between *DJ-1 *and P in the DAGs below.

Generally, this is a plausible assumption provided that the exposure does not modify the genetic structure [57]. In this specific example, this assumption is likely to be true. However, with other environmental exposures this assumption does not hold. For example, the association of some lifestyle factors with genotypes predisposing (or causing) Parkinson's disease is possible as the dopaminergic system is involved in rewarding mechanisms and it is hypothesized to influence some seeking behaviours and addiction (i.e., smoking or alcohol drinking) [58].

The idea of I as a variable to represent interaction is similar to the sufficient component cause (SCC) variables in VanderWeele and Robins [59]. We feel however that our approach presents a few advantages over the SCC framework. As we do not need to incorporate all the sufficient causes (we are not using a causal DAG), the structure of our DAGs is less cumbersome. Also, although for the sake of simplicity we have defined I in terms of binary exposures, we can easily extend it if we are considering multi-valued or continuous exposures. The DAG in Figure 4 shows a complex situation we can imagine, given assumptions 1 and 2, in which there is confounding between both the exposure to paraquat and the disease outcome (Cp) as well as confounding between *DJ-1 *and the disease (Cd), and no other variables are postulated. Confounding between both exposure to paraquat and the disease might be due, for example, to the fact that people exposed to paraquat may also be more likely to smoke, a factor that is negatively associated with the risk of Parkinson's disease [60]. Confounding between *DJ-1 *and the disease might be due to the involvement of the dopamine-mediated rewarding system [58]. Any observational study -any study of these issues in humans- is unlikely to observe all potential confounders. Nevertheless, just to simplify our model, we also assume that:

3. There are no further confounders between either the gene and the outcome or the exposure and the outcome. This is represented by the absence of additional variables and corresponding directed edges in the DAGs below.

Now we turn our attention to looking at the case by evaluating the plausibility of a few different GEI scenarios. As mentioned above, these are loosely based on Khoury et al. [55]. For each of the models that we consider below, we present a more formal description in Appendix 4.

### Model I

**Figure 5:**
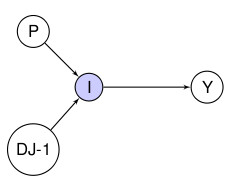
**Both DJ-1 gene and pesticide exposure need to be present to activate the interaction**.

Both exposure and genotype are required to increase risk as in Figure 5. Here, if I is "on" then there *is *an association between the disease and the genetic exposure and the environmental exposure to pesticides when both are present. If on the other hand I is "off" then there is no association -in other words, Parkinson's is only associated with *DJ-1 *and paraquat exposure through the interaction itself. This is an extreme form of interaction that is unlikely to occur in the pathogenesis of common diseases. Does this model describe the relationship between *DJ-1*, exposure to pesticides and Parkinson's disease? For this to be the case, all the Dutch and Italian families with the variant *DJ-1 *and Parkinson's would also have to have been exposed to pesticides. Further, the incidence of Parkinson's amongst the families with the gene variant would have to be the same on average as that of those without the gene variant (if unexposed to pesticides). Similarly, those exposed to pesticides would have to have the same incidence as those not exposed to the pesticides without the *DJ-1 *variant. This is clearly not the case.

### Model II

**Figure 6:**
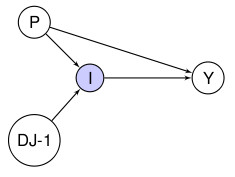
**Pesticide has an effect but DJ-1 only has an effect if pesticide exposure is present**.

The exposure to pesticides increases the risk of disease but the presence of the gene variant alone does not increase the risk of disease, although the variant further increases the risk of disease in the exposed population (Figure 6). In this model, I is switched on and off by P. When P = p* (exposure to pesticides) I = 1, indicating that the interaction is switched "on" and the presence of the variant in *DJ-1 *and Parkinson's is influential. When P = p then I = 0 and whether *DJ-1 *is the variant or wild-type form makes no difference to the outcome Y. It is possible that in some cases exposure to P is protective; i.e., I would take the opposite value of P in a binary situation. In more complex situations, the effect of P might be such that only certain values of P result in interactions and in these cases the values of I and P would not be the same. In this instance, we have that Y depends directly on exposure P; however, Y depends on *DJ-1 *only through the interaction *and *the exposure *when this is present -*i.e. when P = p*.

This model is also not a plausible description of the relationship between the three variables based on the evidence at hand, as it would mean that all the families with the variant and Parkinson's would have to also have been exposed to pesticides.

### Model III

**Figure 7:**
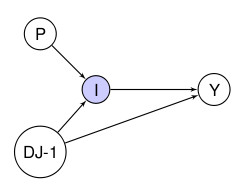
**DJ-1 has an effect but pesticide only has an effect if the gene mutation is present**.

Exposure to pesticides exacerbates the effect of the gene variant but has no effect on persons with the normal genotype. In this model, I is switched on and off by *DJ-1*. The model does not provide either a plausible explanation of the available evidence (Figure 7).

### Model IV

**Figure 8:**
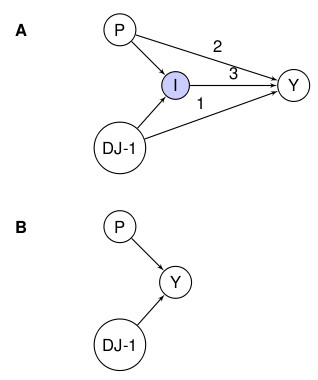
**Both DJ-1 and the pesticide have an effect and there is a possible interaction in A but not in**.

The environmental exposure and the gene variant both have some effect of their own but together they further modify the effect of the other. Here I is a function of *both *P and *DJ-1 *and is defined as follows: I is "on" if and only if both P and *DJ-1 *are "on" otherwise I is "off". Here there are also direct associations between P and Y and *DJ-1 *and Y other than through I; this indicates that there are effects of P on Y irrespective of *DJ-1*, and effects of *DJ-1 *on Y irrespective of P. From the data we cannot distinguish between DAGs A and B in Figure 8.

A core issue with these models is that I is essentially unobservable in humans living under normal conditions; these biological interactions can only be tested in animal experiments. Thus, in humans we cannot disentangle the two DAGs above apart without further information (VanderWeele and Robins [61] provide some tests to determine which individuals present Y only when the interaction I is "on" provided there is no unmeasured confounding). In order to be able to fully tell them apart, an experiment can be conducted or the relative risks can be compared (see Appendix 1).

In light of the evidence on Parkinson's disease, we have to favour one of the two models IV above the other three, as it would appear that both the genetic and the environmental exposure have separate (independent) effects on the risk of Parkinson's. However, from the data on humans we cannot distinguish between the two "type IV" models until we run a study to determine the presence of an interaction. In the case of the Drosophila experiments (see section below) the interaction model on the left-hand side provides a better explanation, as flies with the mutation that have been exposed demonstrate further sensitivity to exposure to pesticides than those who do not have the mutation.

The example we have shown exemplifies, we think, a common situation concerning the interaction between metabolic genes and environmental exposures (e.g. arylamines and NAT2, PAH and GSTM1 and many others) but has the peculiarity that experiments in Drosophila have been done (see below).

### Experimental evidence: the case of the Drosophila

The DAGs above alone cannot be directly used for causal inference unless additional assumptions are made or experiments conducted. The reason is the limited information on potential confounders (and intermediate variables, etc.) that can influence the relationship between the three observed variables. For the sake of making the DAGs clear, we have assumed that there are no confounders; however this is unlikely to be the case in practice as Parkinson's is a multifactorial disease. The method we have proposed can however be extended to include confounders and intermediate variables.

In the case of Drosophila the situation is simpler. Meulener et al. [49] show that both exposure to pesticides and the mutation of *DJ-1 *may be associated with increased risk of neural degeneration. Further, the combination of the two has also been demonstrated to aggravate the condition, as the flies which had the *DJ-1 *gene knocked out exhibited a ten-fold increase in sensitivity to paraquat (which would indicate a supra-multiplicative interaction).

**Figure 9:**
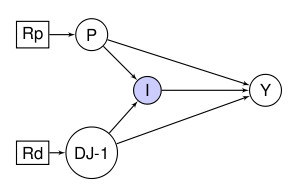
**DAG representing the fruit-fly experiment where interventions were performed both on the genetic make-up and the pesticide exposure**. The interaction can therefore be identified.

As in this case both the genetic make-up and the exposure status of the flies have been intervened upon under controlled conditions, we can make causal inference based on this data by introducing randomisation variables into our DAG. The DAG in Figure 9 is an augmented DAG [38] that includes randomisation variables Rp and Rd. These tell us whether P or *DJ-1 *are being randomised or not and allow us to make inferences about interventions and, hence, causality using DAGs. For a more detailed discussion see Appendix 2.

The DAG in Figure 9 implied that for the Drosophila at least we can state that exposure to pesticides causes an increased risk of neural damage, as does the presence of the mutated *DJ-1 *gene. Also as the combined presence of the mutation and paraquat further increases the risk of neural damage, we can ascertain the presence of an interaction. It should be noted that DAGs do not specify or constrain the model of statistical interaction, which can follow either an additive or a multiplicative null hypothesis model.

In the case of humans, we cannot assume such randomisation variables exist (except in Mendelian randomisation which, however, applies to gene variants only, and not to exposure); thus, we cannot expand the DAG in Figure 6. On the other hand, etiologic factors and clinical phenotypes are usually more diverse in human diseases than in animal models; inferences to human diseases from relatively simple animal experiments have well known limitations. An avenue for progress lies in integrating DAGs with the inductive reasoning implicit in Hill's guidelines.

### Application of causal guidelines to DJ-1 and exposure to paraquat for Parkinson's disease

Following the DAG approach, we established the relationship between genes and some environment exposures in promoting Parkinson's disease, and we proposed different interaction models between *DJ-1*, pesticides and Parkinson's disease. In order to apply Hill's causal guidelines to the DAGs we are going to work with (Figure 6A), we need to label each of the edges. Throughout the rest of this section we use the following labels:

• The edge between *DJ-*1 and Parkinson's disease is referred to as [edge 1],

• The edge between exposure to pesticides and Parkinson's disease is referred to as [edge 2],

• The interaction between *DJ-1 *and the exposure to pesticides in causing Parkinson's disease is called [edge 3].

Hill's guidelines are discussed in a slightly different order than in the original version and statistical significance is omitted because it refers to the contingent evaluation of each study and does not require a specific discussion in relation to genomics.

(a) **Strength of association. **DJ-1 has been seen to be lacking in Dutch families with Parkinson's disease, and to be functionally inactive because of a point mutation in the Italian families studied by Bonifati and cols [51]. The deletion showed complete cosegregation with the disease allele in the Dutch family [51]; also in the Italian family the homozygous mutation showed complete cosegregation with the disease haplotype, and absence from large numbers of control chromosomes [62]. Although the function of the DJ-1 protein is unknown, these data suggest a strong association between the *DJ-1 *gene and the occurrence of Parkinson's disease in certain families [edge 1]. To establish the strength of the association between specific environmental factors and a disease is far more complicated, mainly due to the quality of exposure assessment, the latency period, and body concentrations during the lifecourse. A meta-analysis of the association of pesticides and Parkinson's disease points out that both pesticide exposure in general and selective exposure to paraquat seem to be associated with Parkinson's disease, with odds ratios ranging from 1.25 (95% C.I.: 0.34 - 4.36) to 3.22 (95% C.I.: 2.41 to 4.31) [53] [edge 2]. With respect to the interaction parameter, there is as yet no epidemiological study that has tested whether there is an interaction between *DJ-1 *and pesticides; thus neither the existence nor the strength of such an association are known. However, knockout models of Drosophila Melanogaster (fruit fly) lacking DJ-1 function, display a marked and selective sensitivity to the environmental oxidative insults exerted by both paraquat and rotenone [49], suggesting an interaction between these toxicants and the *DJ-1 *genotype [edge 3] in animal models and, consequently, that in humans the interaction between the chemicals and *DJ-1 *is biologically plausible (as can be seen, Hill's criteria often "interact", i.e., they are often related to each other, as in this paragraph the strength of association is related to the biological plausibility).

(b) **Consistency of the association. **After the first variants described, different variants of the *DJ-1 *gene associated with the same Parkinson's disease phenotype have been found in patients of Ashkenazi Jewish and Afro-Caribbean origins [63,64] [edge 1]. The association of paraquat and rotenone with Parkinson's disease is more consistent in animals (in which these two toxicants are often used to produce animal models of the disease) [54] than in humans. In environmental epidemiological studies in humans, the association has been found substantially consistent across studies, although some associations did not reach statistical significance, mainly due to limited sample size. In a study in Taiwan, where paraquat is routinely used in rice fields, a strong association between paraquat exposure and Parkinson's disease was found; the hazard increased by more than six times in subjects exposed for more than 20 years [64]. A dose-response curve with length of exposure was also observed in plantation workers in Hawaii [65], and British Columbia [66]. In a population-based case-control study in Calgary, occupational herbicide use was the only significant predictor of Parkinson's disease in multivariable analysis [67]. However, in another population-based case-control study in Washington, the odds ratio of 1.67 did not reach statistical significance (95% CI: 0.22-12.76) [68] [edge 2]. There is yet no evidence from human studies to confirm the consistency of GEIs in the causation of Parkinson's disease [edge 3]. Furthermore, genes other than *DJ-1 *may be involved in the etiopathogenic process, and so may be exposures other than pesticides, and other GEIs. Since environmental conditions vary substantially across the globe, and the role of one gene, one exposure or one GEI is often dependent on other genes, exposures and GEIs, lack of consistency is to be expected in studies conducted in different settings, and in particular when studies focus only on a few GEIs and overlook other interactions.

(c) **Specificity of the association. **The specificity of the association between *DJ-1 *gene mutations and Parkinson's disease [edge 1] will be clearer once the data on the pathological features of the *DJ-1 *patients will be available (see Appendix 3). Chronic systemic exposure to rotenone has been demonstrated to cause highly selective nigrostriatal dopaminergic degeneration associated with characteristic movement disorders in rats [54] [edge 2]. Similarly, paraquat caused a significant loss of nigral dopaminergic neurons in mice compared to controls [69] [edge 2]. Once an appropriate epidemiological study is set up aimed at studying GEIs in this context, results from the pathological analysis of the sample subjects will help to answer important questions regarding the aetiological pathway of the disease [edge 3].

(d) **Temporality. **This criterion does not apply directly to genotype, as it is determined at conception and it remains constant over time (see Appendix 1) [edge 1]. However, temporality is crucial if we go beyond genetic effects and consider epigenetic mechanisms; e.g., gene regulation by environmental factors [14,16-18]. This problem goes beyond the present contribution, but is worth mentioning. Concerning pesticides, temporality might be a concern given that all studies on GEI in Parkinson's disease are case-control studies, which are particularly prone to selection bias, disease progression bias, and so-called "reverse causality" [3,70,71]. In this case, while it is unlikely that suffering from Parkinson's disease would have influenced past exposure to pesticides or their metabolism, it could have influenced recall. The observed dose-response relationship, with 20 years of exposure required [53], favours the existence of a true association, and is compatible with disease characteristics of neurodegeneration, making the temporality pattern suggestive of a causal role [edge 2].

(e) **Biological gradient. **This criterion does not apply since we are dealing with a recessive model of inheritance. Nonetheless, a co-dominant model should not be completely ruled out as a careful neurological evaluation of heterozygote subjects might point out some sub-clinical changes [edge 1]. A dose-response relationship between toxicant exposure and neural loss in animal experiments has been observed [72]. In addition, several studies observed a positive correlation with duration of exposure to, and high dose of, herbicides and insecticides in humans [53] [edge 2].

(f) **Biological plausibility. **Biological plausibility of the *DJ-1 *mutation awaits the discovery and characterisation of the encoded protein [edge 1]; the capability of some toxicants to induce a progressive cellular loss in the substantia nigra and to be responsible for a progressive clinical syndrome with an intervening latent period has been hypothesized [54] [edge 2]. It is, therefore, plausible that these two factors may interact during the course of life producing Parkinson's symptoms in genetically susceptible individuals [edge 3].

(g) **Coherence with previous knowledge. **Confirmation of the presence of different mutations on the same *DJ-1 *gene in families with other background origins but manifesting the same symptoms supports the involvement of the gene in the disease [63,64] [edge 1]. A role of herbicides in neurodegeneration has also been studied with generally confirmatory results [edge 2].

All these considerations taken together suggest that there may be a potential interaction between exposure to certain pesticides and the *DJ-1 *mutation in the risk of developing Parkinson's disease. However, as no studies on humans have yet been specifically conducted to investigate this issue, we can use the evidence only as a reason to further explore this interaction, perhaps by conducting a more targeted study. As mentioned, it is likely that other factors (both genetic and environmental) also contribute to the final development of the disease.

In the example above we have shown that the DAG approach can be complemented by the use of Hill's guidelines when no experimental evidence can be brought to bear on a particular gene-environment interaction.

## Conclusions

While medical and epidemiologic evidence is routinely assessed to determine the causal nature of relationships involving personal and environmental causes of disease, genetic associations have so far not undergone similar scrutiny. However, like epidemiologic studies, genetic studies are also commonly based on observational studies, and may thus be affected by similar weaknesses. As the contribution of genetics to the understanding of disease etiology becomes more important, causal assessment of genetic and genomic evidence will become a key issue [73].

We have explored two complementary ways to tackle causality in gene-environment interactions. The application of causal guidelines to genetics is not straightforward, and it becomes very complex, in particular, if one wants to study gene-environment interactions, as we have illustrated with Parkinson's disease. Hill's criteria were developed to examine one factor at a time and have seldom been applied to evaluate the causal nature of complex relationships involving several exposures. On the other hand, graphical approaches like DAGs are effective in making potential causal networks explicit, but are insufficient to establish the strength of evidence (e.g., edges cannot be interpreted as causal without some kind of additional external support). This seems to be a general problem of causal networks, not only gene-environment interactions.

The graphical approach is useful in particular for clarifying complex causal pathways. We have applied it to a simple example where the inner workings (i.e., the detailed biological mechanisms in animal models) of the interaction are not completely known. The approach we propose uses the statistically formal representation of DAG models. This is in contrast to Weinberg's paper [74] which, although invaluable in highlighting the pros and cons of DAG models, does not actually use DAGs, but heuristic diagrams not dissimilar to those proposed by Ottman [56] and, over 35 years ago, Susser [75]. In the approach advocated by VanderWeele and Robins [76], DAGs are considered implicitly causal. We feel that this can be overly confident when the bases for inference are observational studies, which is generally the case in human genetic studies. Thus, we propose a more conservative approach that involves assessing the causal properties of each individual relationship.

A final caveat to interpreting DAGs involving genes as causal is whether genetic variants can be considered causes of diseases [30]; in a strict sense this issue is unresolved. It is generally accepted that the causal nature of a relationship can be assured when interventions (such as those performed in experiments) take place. This is because controlled interventions usually (and more easily) guarantee that the association investigated is not confounded (but this is not an absolute rule). VanderWeele and Robins [61,76] assume that genes can be considered causes of diseases, without discussing the implications or bringing additional information such as Hill's criteria into play; we believe that this is a strong assumption: knowledge on the mechanisms that govern the subclinical development and clinical course of complex diseases is rather limited.

In summary, we believe that the DAG and causal criteria-based approaches can complement one another, as one helps to assess the strength of evidence, while the other disentangles -in a visual but also formal way- the role played by genes, environmental exposures, and their interactions. The method we suggest can easily be extended to more complex situations and in particular to the understanding of gene-gene associations and interaction. The problems we raise are likely to become more relevant as genome-wide association studies provide new candidate genes for a variety of diseases, Mendelian randomization is used to assess exposure-disease associations, and gene-environment interactions are further investigated in genetics and epigenetics.

## Competing interests

The authors declare that they have no competing interests.

## Authors' contributions

SG participated in developing the graphical and statistical aspects of the approach and methods proposed in the paper. VG participated in developing the approach and method proposed in the paper and applying it to the case of Parkinson's. MK participated in the development of the approach and method proposed in this paper and advised on its relevance to gene-environment interactions and epidemiology. MP participated in the development of the approach and methods proposed in this paper and advised on its relevance to molecular genetics and epidemiology. VP participated in the development of the approach and method proposed in the paper with particular focus on how to combine the graphical and causal Bradford-Hill criteria in the context of genetic causation.

## Appendix 1 Using Austin Bradford Hill's guidelines in genetics and genomics

There are some general aspects to consider when tackling cause-effect relationships in genetics. First, most associations for individual genetic variants and common chronic diseases have weak to modest effects. Empirical findings show that even for fairly well established associations, the effect sizes are weak to modest; i.e., relative risks are usually under 2, and often between 1.2 and 1.6) [11]. Generally speaking, the stronger the association between a risk factor and a disease, the more likely it is that the association is causal, because confounding and other biases are unlikely to explain it away. However, in genetics the penetrance of an individual genetic variant associated with a disease depends on the interactions of the variant with external exposures, the internal environment, or other genetic variants. In spite of the etiologic complexity of common diseases and the resulting weak effects of individual genetic variants, theoretical work suggests that the combination of as few as 20 common variants with weak to moderate effect sizes, when put together as a system of variants (or genomic profiles), can account for a substantial attributable fraction of the disease in the population [12]. On the other hand, a large number of rare variants each contributing (or causing) a strong disease risk may also be a plausible explanation. The potential rarity of highly-penetrant variants, the weakness of common associations, and the frequency of complex gene-environment interactions pose severe challenges to the statistical power to find marginal effects of single gene variants on risks for common diseases. In fact, the strength of the association with the gene (main effect) may be low while the gene-exposure interaction is strong. This may be more convincing evidence of the truly causal nature of the association, given the available biological knowledge on environmental influences on gene expression.

Consistency in genetic studies was traditionally poor in the "candidate gene" era, with few associations confirmed in more than one study [13], but this has changed rapidly with genome-wide association studies (GWAs). More than 600 stable replicated hits have been reported in 2007 and 2008 from GWAs, due to an in-built, strong process of replication of findings. One advantage of GWAs is that they are published only if the results are replicated in 3-4 or more independent studies. As a result in genetic epidemiology there is now a widely accepted requirement for "internal" consistency. A similar approach would be invaluable in non-genetic epidemiology but is currently not practiced. Poor replication for candidate genes is related to multiple factors, including type 1 errors ("false positives") and publication bias, as well as to methodological issues as biases in the selection of cases and controls, exposure assessment errors, and confounding.

In addition, the expression of genes is so dependent on the surrounding circumstances (other genes, internal environment -e.g., immunological and nutritional status [14]-, external physical environment, gene expression), that the same main clinical effect of a gene variant is difficult to capture in different studies conducted under different conditions. In fact, such main effects may not be identical in different studies that are conducted in actual -sometimes, very different- human contexts; a genuine heterogeneity of human genetic effects across population groups -and individuals- is to be expected on the basis of knowledge on how biological, clinical and environmental processes jointly cause disease in humans. An example of the influence of study design is the investigation of gene-disease associations in founder populations, in which the effect of a genetic variant is likely to be higher than the average across all populations [15]. Another example is familial aggregation studies, where familial disease risks are influenced not only by the genetic mutations or variants of interest, but also by other genetic and epigenetic processes; if the latter are overlooked, the penetrance of the former may be overestimated [16-18].

To some extent it is reasonable to hope that genetic associations are specific, thus facilitating causal inference. For example, 5-HTT variants have been associated specifically with bipolar disorder, probably because of the role of the gene in serotonin metabolism [19]. But expectations of specificity may disregard biological knowledge (e.g., on cofactors, multiple causes and effects) that makes unspecificity more plausible. A potential problem in the use of specificity as a criterion for causality is that many genetic variants belong to metabolic, inflammatory, homeostatic and other pathways that could influence multiple disease processes. This is an extension of the concept of pleiotropy that we see in single gene disorders. For example, MTHFR variation involves folic acid and methylation pathways that may have potential relevance to the genesis of many disease outcomes, as birth defects, cardiovascular disease and cancer [20]. The same is likely to be true for DNA repair genes [21]. This issue has long been observed in non genetic epidemiology in relation to some common risk factors, such as socio-economic status or cigarette smoking, which are associated with many disease outcomes. The value of specificity increases with increasing knowledge about the constituents of the exposure (e.g., PAHs and other carcinogens for cigarette smoking), and of its biological or environmental effects. For example, on the basis of functional knowledge, only bladder cancer, and perhaps colon cancer, may be expected to be associated with NAT2 variants [22-24]. Such postulated associations are biologically plausible because there is evidence that aromatic amines or heterocyclic aromatic amines, which are metabolised by NAT2, are involved in bladder or colon carcinogenesis. Nevertheless, NAT2 associations are also observed with breast and lung cancer and mesothelioma [25,26], without evidence of biological plausibility. This unexpected non-specificity may be true and due, for instance, to a pleiotropic effect of the exposure; or the apparent association with the outcome (in this case, other than bladder cancer) may be confounded by yet unknown factors. Similar situations are encountered in clinical medicine and non genetic epidemiology; for example, the early observation of an inverse association between hormone replacement therapy (HRT) and mortality due to accidents and violence, which was of the same magnitude as that originally found for cardiovascular mortality [27]. This prompted a debate on the causal nature of the association between HRT and cardiovascular mortality, as no plausible biological reason for the protective effect of HRT on violent death could be argued.

Temporality is also relevant to the study of the genotypes; since gene variants are inherited and do not change after conception, they precede the onset of disease indeed. In addition the temporal pattern with which a particular variant/mutation manifests itself can be relevant. In Huntington's disease, for example, there is the phenomenon of "anticipation" (younger age of disease onset in one generation than in the previous) depending on the number of the repeated triplets in the gene (which tend to increase in the offspring). For acquired genetic alterations (e.g., somatic mutations) temporality is also important; in persons living in normal conditions the timing of occurrence of the mutation often cannot be observed directly. A collection of archived specimens may help, as can knowledge on the usual course of events gained from molecular pathology studies. For epigenetic mechanisms temporality is even more crucial, but it is beyond the purpose of this article [14,16-18].

In genomics, the possibility of observing a dose-response gradient depends on the model of genotype-phenotype relationships. Even for a diallelic system at one locus, there could be recessive, dominant or codominant models. The biologic model for the action of numerous alleles at different loci is more complex and is essentially unknown for most common diseases. Only if the genetic model is codominant can a dose-response be observed. However, a different kind of dose-response is observable if we consider the cumulative effect of multiple genes or SNPs. Both the risk of lung cancer and the levels of DNA damage can increase approximately linearly with an increasing number of "at risk" gene variants [21,28]. Gene copy number variation can lead to more complex dose-response relationships. Quantitative continuous markers used in epigenetics (promoter methylation) and transcriptomics (gene expression) may be analyzed in search of dose-response effects (linear or non-linear).

In genetics experimental evidence comes mainly from animal studies in which knock-out organisms are used in order to have a pure genetic disease model. This directly tests the effect of the absence or presence of specific genetic factors on the organism. Extrapolation of the results of these experiments to humans is challenging due to differences between humans and the knock-out organisms in both the genetic make-up and the potential types of gene-gene and gene-environment interactions. Genetic experimental studies have also long been known to reproduce disease phenotypes (e.g., in mice) that are only a partial approximation of the complex human disease; an example is the Super Oxide Dismutase-1 (SOD1) mutated mouse model for Amyotrophic Lateral Sclerosis (ALS), which has different motor characteristics than the human disease [29].

## Appendix 2 The calculus of the Decision Theoretic Framework (DTF)

### The calculus of DTF

Conditional independence [12] is the tool DTF uses to *a) *express how variables are associated and *b) *to understand when it is possible to make inferences about causal associations from data that are observational. It is best described as follows: consider 3 variables A, B and C. Say that *Pr(A,C|B) = Pr(A|B)Pr(C|B) *(where Pr(.) means probability of).

Then we can say that A is independent of C given B - formally: *A *┴┴C|B.

This means that if we know what B is, knowing what A is gives us no further information on C; e.g., if we want to know the genetic make up of Alfred (A), we can gain some information by looking at his brother Colin (C). If however, we can see their parents Barry and Barbara (B), then knowing about Colin gives us no further information on Alfred. This shows where the "familial" terminology used in DAGs comes from.

Conditional independence is a non-graphical (and non-causal) equivalent of the d-separation criteria used in the causal DAG approach [11]. It forms the basis for the formal treatment of DTF, and its manipulation allows us to determine under what circumstances we can equate the results of observation to those of experiment [35,44].

The original role of DAGs in the statistical literature is to encode statistical associations (described, for instance, by Chi-squared tests). Thus, in DTF the lack of directed edges in a DAG is viewed as conditional or marginal independence between variables, not a lack of a causal relationship. There are two problems with interpreting DAGs encoding such associations as causal. The first problem is that often there is more than one DAG representing the same set of conditional independences (see example below). To determine which, if any of them, is causal, we must use knowledge that is not inherent in the data or the DAG (e.g., time ordering). The second problem is that we often do not have data on all the variables that play a role (causal or otherwise) in the problem we are considering. This means that the DAGs only tell us about the relationships between the variables we have observed, making a causal interpretation dangerous.

Consider the following simple example: A and B are proteins produced in the body and C is a cancer thought to be associated to the production of A and B. It is possible to artificially increase the amount of B in the system and we would eventually like to know whether this could prevent the emergence of the cancer C. However, at this point we do not know whether A or B are produced by the presence of C or indeed whether there is any natural ordering to the appearance of the three variables. We obtain the conditional independence A┴┴C|B from data on a number of individuals in a case control study investigating possible causes of C. This is encoded by all three DAGs in the Figure 2. These three DAGs only tell us one thing, namely that the cancer is not directly associated to protein A (when we only consider these three variables and the individuals in the study). They do not tell us whether treating patients with B will have a positive effect on the incidence of C or indeed how A and B are associated. Thus, trying to determine whether intake of B will act as a preventive agent (i.e., whether B causes C) based only on current knowledge and the DAGs is impossible. When we face a problem that we do not understand fully, interpreting one DAG or even one particular directed edge as causal can be difficult.

### Randomisation and interventions

One way of determining whether relationships depicted in a DAG describing observational data are causal is to relate it to an equivalent situation under intervention or randomisation. It is generally accepted that the ideal for causal inference is the randomised controlled trial because confounding is eliminated or attenuated. It is generally also accepted [36] that when we perform an external intervention, such as randomisation on a system in equilibrium, we can view the consequences as causal. Thus, intervention is a formal way of asserting cause-effect relationships.

In DTF we introduce randomisation as a variable R (Figure 3). To clarify, consider the following example. Assume that X is a binary variable that can be forced to take on a particular value or "set". It takes on two values: "active" (*X *= *a*), or "baseline" (*X *= *b*). The randomising variable R has the same settings as X as well as the observational setting *R*= Ф (the empty set). When *R *= *a *then *X *= *a *with no uncertainty (imagine forcing X to take on this value, say by administering the treatment to a compliant patient). Similarly, when *R*= *b, X *= *b *with no uncertainty. Finally when *R *= Ф, X is allowed to arise without intervention and can take on the values *a *and *b *as in an observational study. For causal inference in DTF we want to estimate (usually the expected value of) the outcome Y given that an intervention has happened. For example, if we want to know which treatment, active or baseline, is better for Y, we might look at the difference in the expected value of Y given these treatments: *E(Y *| *R *= *a*)- *E(Y|R *= *b*). This would then be a measure of the causal effect of *a *vs *b*. In observational studies, we do not have *E(Y|R *= *a*) the *interventional expectation*; rather, we have *E(Y| X = a, R *= Ф) the *observational expectation*; similarly for *b*. The question is, therefore, how to make an inference about the former using the latter. One assumption that is often made is that all observed confounders U are observed. However, this is often not possible and other approaches that *simulate *randomisation, such as the instrumental variable approach known as Mendelian randomisation [37] can be used. See Dawid [41], Didelez [42], and Geneletti [43] for formal examples.

Introducing randomisation can also help us distinguish between intermediate variables and confounders, as when X is randomised the association between X and any confounders U is severed, whilst that with intermediates is not (A and B in Figure 3). Statistically, if after randomising X the distribution of U conditional on X remains the same as before randomisation, then U is a confounder rather than a mediating variable, as this means that U is independent of X when it is randomised. This corresponds precisely to the situation described by the DAG in Figure 3A. If U depends on X then we have that U is a mediator as in Figure 3B.

As also shown in Figure 3B, interventions are represented by decision nodes (square boxes) in augmented DAGs [38], and these can be used to make some causal inferences, as DAGs explicitly represent interventions. By introducing the randomisation/intervention variables explicitly into the DAG, we can use conditional independences to determine when it is possible to estimate the causal effect (based *R *= *a,b*) from data that are observational (based on *R *= Ф and *X *= *a,b*). Again, as a detailed description of the formal DTF is beyond the scope of this paper, we refer the interested reader to previous work [41-44].^42 43^

## Appendix 3 Parkinson's disease: environmental and genetic risk factors

### Parkinson's disease: environmental factors

Large epidemiological studies aimed at identifying risk factors for Parkinson's disease have suggested a role of 1-methyl-4-phenyl-1,2,3,6-tetrahydropyridine (MPTP) (a compound accidentally produced in the manufacture of illegal drugs), of some pesticides, of certain metals and of polychlorinated biphenyls [46]. On the other hand, tea and coffee drinking, use of non-steroidal anti-inflammatory drugs, and high blood levels of uric acid have been suggested to be protective for Parkinson's disease [46].

### Parkinson's disease: single gene disorders

To date, eleven monogenic forms have been identified (with *PARK1 *to *11 *gene acronyms); they will be selectively discussed below (Table 1) [47]. However, monogenic forms of Parkinson's explain no more than 20% of the early-onset cases of the disease, and less than 3% of the forms with onset in the old ages, a situation that is common to many chronic diseases as breast cancer (e.g., role of BRCA1) or heart disease (e.g., Familial Hypercholesterolemia). Most forms of the disease appear to be caused or at least influenced by complex interactions between several genes, or between genes and environmental factors.

**Table 1:** Main identified genes involved in Parkinsonism, with their biological, clinical and pathological main features

Gene (locus)	Protein	Function	Inheritance	Pathology	Clinical phenotype
*1SNCA *(PARK1/4)	α-synuclein	Signal transduction, membrane vesicle trafficking, and cytoskeletal dynamics	Dominant	Diffuse Lewy bodies (prominently nigral and hippocampal neuronal loss)	Early onset progressive L-Dopa responsive Parkinsonism, cognitive decline, autonomic dysfunction and dementia

*LRRK2 *(PARK8)	Dardarin	Cytosolic kinase with several functions (including substrate binding, protein phosphorylation and protein-protein interactions)	Dominant	Predominantly Lewy bodies disease (rare cases with neurofibrillar tangels and/or nigral neuronal loss	Parkinsonism consistent with sporadic Parkinson's Disease. Dystonia, amyotrophy, gaze palsy and dementia occasionally develop

*PRKN *(PARK2)	Parkin	E3 ligase (conjugating ubiquitine to proteins to target them for degradation by the proteasome)	Recessive (rare "presudo-dominant" cases reported)	Predominantly nigral neuronal loss (compound heterozygotes with Lewy bodies or tau pathology are described)	Early onset Parkinsonism, often presenting with dystopia, with diurnal fluctuations. Typically responsive to very low doses of L-Dopa

*PINK1 *(PARK6)	-	Mitochondrial kinase	Recessive	Undetermined	Early onset Parkinsonism, slowly progressive and responsive to low doses of L-Dopa

*DJ-1 *(PARK7)	-	Oxidative stress signalling molecule on mitochondria	Recessive	Undetermined	Slowly progressive early-onset Parkinsonism occasionally with psychiatric disturbances; rare compound heterozygotes with Parkinsonism and dementia or amyotrophy are described

The α-synuclein, encoded by the *SNCA *gene, is a protein with several functions in signal transduction and vesicle trafficking; it is also a competitive inhibitor of an enzyme involved in the L-Dopa biosynthesis. Three known dominant mutations on the *SNCA *gene have been identified in families affected by Parkinsonism with dementia characterised pathologically by diffuse Lewy bodies, mainly composed of α-synuclein. The identification of these mutations contributes to the contention as to whether the so-called Lewy body disorders (Parkinson's disease, Parkinsonism with dementia, and dementia with Lewy bodies) represent a continuum or have to be considered as distinct diseases [47]. This is thus as well an excellent example of a situation in which researchers try to elucidate the causal relationships between a complex set of genotypes and a rich spectrum of clinical phenotypes.

The *LRRK *gene encodes for a protein involved in multiple functions; three dominant mutations are known. Pathologically, the disease is characterised by a typical Lewy body pattern consistent with the *post mortem *diagnosis of Parkinson's disease. However, some cases with tau-positive pathology without Lewy bodies have been observed even within the same family. The pathway leading to one or the other condition is likely to be influenced by genetic and/or environmental factors that remain to be identified [47].

There are more than 50 known variants in the *parkin *gene and their effect on the disease appears to be recessive. Subjects with homozygous mutations leading to complete loss of *parkin *expression are found to have a selective loss of dopaminergic neurons in the substantia nigra and in the locus coeruleus without Lewy bodies or neurofibrillar tangles. However, subjects with compound heterozygous mutations (a diploid genotype in which two copies of a gene carry different mutations) may present pathologically with Lewy bodies or neurofibrillar pathology. This behaviour can be due to the fact that the outcome is mutation-specific: some mutations can reduce rather than abolish the protein activity affecting substrate specificity. Otherwise, these two different outcomes can share the primary cause (as for the *LRRK *case), which is subsequently influenced by gene-gene and/or gene-environment interactions [47].

For the last two recessive mutations, *PINK-1 *and *DJ-1 *there is no pathological information available. The protein encoded by *PINK-1 *gene is a mitochondrial kinase that seems to be involved in protecting the cell from mitochondrial dysfunction and stress-induced apoptosis [47]. The protein encoded by *DJ-1 *gene also is localised on mitochondria, but it seems to belong to the chaperones family, induced by oxidative stress [48]. This protein has been demonstrated to be involved in cell protection during oxidative stress. Intriguingly, reduced *DJ-1 *expression in *Drosophila melanogaster *results in susceptibility to oxidative stress and proteasome inhibition, which leads to a selective sensitivity to the environmental chemical agents paraquat and rotenone [49].

## Appendix 4 The gene-environment interactions (GEI) models in formal termsº

Below is a more formal treatment of the GEI models we consider in the main text. In addition to considering the conditional independence statements we also look at the observed relative risks as these can give us information about the type of interaction we are dealing with. We assume throughout that the interaction is synergistic rather than antagonistic and also that the appropriate monotonicity conditions between risks hold.

First the assumption of no dependence between genotype and exposure is given formally *DJ *- *1 *┴┴ *P*.

Relative risks are defined as follows:

 denotes the risk of disease of *P *= *p *and *DJ-1 *= *d*, relative to the risk given by  and .

### Model I

In addition to the above assumption the Model I DAG represents the following conditional independence

■ Y ┴┴ (DJ-1, P)| = 0 - this tells us that when either the variant or the exposure are not present, the disease is not associated with the mutation or the exposure.

In terms of relative risks this model implies that

### Model II

In addition to the above assumptions we have:

• which says that P does not affect Y when .

### Model III

The formal assumptions are the complement of those in Model II.

### Model IV

There are no additional assumptions here. In this case the only way to determine which model holds is to run an experiment or an observational study to estimate the effect of the interaction. In this scenario it is essential to decide on the scale of the interaction, as this will determine whether an effect is found or not. In the case of the fruit fly, there appeared to be an increase of risk of neural damage on the log scale, indicating a multiplicative model.
